# SARS-CoV-2 infection is not associated with a higher rate of post-operative complications in adult appendectomy patients in Peru: Cross-sectional study

**DOI:** 10.1016/j.amsu.2021.102582

**Published:** 2021-07-21

**Authors:** Eduardo Huamán Egoávil, Lacey LaGrone, Regina Ugarte Oscco, Sergio Endo Ramos, Alex Diaz Baltazar, César Vergel Cabrera

**Affiliations:** a Surgical Center of the Hospital Emergencia Ate Vitarte (HEAV), Carr. Central 506, Ate, 15491, Lima, Peru; b Emergency and Critical Care Service of the Hospital Nacional Guillermo Almenara, Jirón García Naranjo 840, La Victoria 13, Lima, Peru; c Trauma, Acute Care Surgery, Medical Center of the Rockies, UCHealth, 2500 Rocky Mountain Ave., Suite 2200, Loveland, CO, 80538, USA

**Keywords:** Global surgery, Adult appendectomy, SARS-CoV-2 infection

## Abstract

**Background:**

We present outcomes of patients with SARS-CoV-2 undergoing appendectomy in order to aid in clarification of current controversies regarding safety of therapeutic options for emergency surgical diseases in patients with SARS-CoV-2. Peru has the greatest number of per capita deaths due to SARS-CoV-2 of any country and is one of few with a COVID-dedicated hospital.

**Materials and methods:**

This prospective observational study included all adult patients with acute appendicitis admitted to an urban, public, COVID-dedicated hospital over two months. Baseline characteristics and post-operative outcomes at 28 days are reported.

**Results:**

58 patients, 35 male and 23 female, ages 15–73 years with SARS-CoV-2 as diagnosed by IgM (12%), IgG (19%) or both (69%) and acute appendicitis as diagnosed using the Alvarado Score and confirmed intraoperatively were enrolled. All patients presented with right lower quadrant pain, 86% with leukocytosis, 88% with nausea/emesis and no patients with respiratory complaints. All patients underwent open appendectomy, 90% under regional anesthesia. Average operative time was 54±25 min, length of stay 2.5±1.5 days. 14% of patients had a post-operative complication, all were minor, four (7%) incisional surgical site infections, one (2%) organ space, and three (5%) incisional seromas, no deaths or serious complications.

**Conclusion:**

Open surgical management of acute appendicitis with regional anesthesia in adults with pre-operative diagnosis of SARS-CoV-2 is feasible and not associated with an increased frequency or severity of post-operative complications, longer operative time, or extended hospitalization as compared to reports in similar patients without SARS-CoV-2.

## Highlights

• The SARS-CoV-2 pandemic has caused the collapse of health systems across the globe.
• Acute appendicitis is the most common emergency general surgery pathology.
• Surgical management of appendicitis and diagnosis of SARS-CoV-2 is feasible.
• In low-income countries, we recommend early appendectomy for SARS-Cov-2 patients.
• Recommend cautious generalization of reports on higher perioperative mortality.

## 1 Introduction

The SARS-CoV-2 pandemic has caused the collapse of health systems across the globe, including that of our country, Peru [1]. According to Johns Hopkins University at the time of manuscript preparation, Peru has the greatest number of deaths per capita due to SARS-CoV-2 of any country in the world [2], and our hospital is one of few globally, to be dedicated solely to the care of patients with SARS-CoV-2, and the only we are aware of in Latin America [3]. Since the beginning of the pandemic, the recommendation for surgical systems has been to continue provision of care to the emergency surgical patient [4]. Initial studies indicated an elevated incidence of peri-operative complications in patients with SARS-CoV-2 [[5], [6], [7], [8]] with the associated increased mortality primarily attributed to respiratory complications [8]. Surgical emergencies, and specifically surgical management of acute appendicitis, have decreased in frequency of hospital admission, likely due to patients with early disease being managed without directed therapies in their homes [9].

The initial recommendations of the American College of Surgeons [10] and the Association of Spanish Surgeons [4] recommended patients with SARS-CoV-2 and uncomplicated acute appendicitis are treated ‘conservatively’, with pain management and antibiotics. There are French reports of this approach proving feasible, with lower rates of complications as compared to surgical intervention [11].

There are few studies illuminating surgical complication rates in patients with SARS-CoV-2. The available reports are primarily from countries with health systems which are fundamentally different in resources and challenges from Latin American countries like Peru. We thus elected to conduct an observational study, during two months, in a group of patients with the pre-operative diagnosis of both SARS-CoV-2 and acute appendicitis. These patients were operated on early [12], within 8 h of admission, via an open surgical approach. The operations occurred at an urban hospital which was specifically designated as a Coronavirus (COVID-19) hospital to shore-up the health system during the crisis created by the pandemic in Peru: the Hospital Emergencia Ate Vitarte.

## 2 Methods

Adult patients of either sex with a clinical diagnosis of acute appendicitis who presented to the hospital designated for patients with known COVID-19 in June and July of 2020 were included in this prospective observational study. The Hospital Emergencia Ate Vitarte (HEAV) is a 250 bed hospital with 100 critical care beds, and eight beds dedicated for patients undergoing emergency surgery. There are two operating rooms which are available 24 h per day, seven days per week. The hospital is part of the public health care system within Lima, Peru, a city with a population greater than ten million people.

The Alvarado Scale [13] was used to make a clinical diagnosis of appendicitis prior to the transfer of patients from other health centers to HEAV. The diagnosis and grade of appendicitis was ultimately determined intraoperatively using the Gomes Classification [14]. This classification largely overlaps with the American Association for the Surgery of Trauma (AAST) grading system [15], but unlike the AAST system does not include computed tomography or pathology findings, which were not available for the majority of our patients [15]. Our approach to diagnosis and management mirrored that recommended by the World Society of Emergency Surgery (WSES) in the 2016 Jerusalem Consensus Conference, with initial triage performed via the Alvarado score as mentioned, and subsequent clinical decisions aided with imaging, in our case ultrasound, or observation [16] with the exception of ultimate open, rather than laparoscopic, appendectomy. Post-operative complications were captured during the initial hospitalization and during follow-up via telephone at seven, 14, and 28 days post-operatively. We selected this period of follow-up given reports of post-operative complications primarily presenting within the first week after appendectomy [17].

Investigators used a standardized data sheet to collect this data from the electronic clinical record or in follow-up interviews of patients. Complications were classified according to Dindo-Clavien, which grades events according to their impact on morbidity or mortality of the patient, and additional interventions required to treat [18]; and according to the Center of Disease Prevention and Control (CDC) surgical site infection grading [19].

The diagnosis of infection with SARS-CoV-2 was made pre-operatively using a serologic test identifying IgG or IgM using a rapid assay (Orient Gene®) which has been reported to have a sensitivity of 96% (CI95% 90%–99%) and specificity of 100% (CI95% 93%–100%) [20]. Polymerase Chain Reaction (PCR) testing had very limited availability in the country at the time and was not available for clinical use at the study or referral hospitals. The process of admission, use of personal protective equipment, anesthesia administration and surgical intervention were accomplished according to the local institutional guidelines: Program for Safe Surgery in COVID-19. The local institutional guidelines were developed in HEAV and are pending registration with the National Institute for the Defense of Competition and Protection of Intellectual Property (INDECOPI).

Means with standard deviations were calculated for the out-of-hospital pre-operative time (from time of diagnosis at another health center until admission to HEAV) in hours, operative time in minutes, and hospitalization in days.

The study was authorized by the Hospital Administration within the project: Results of Emergency Abdominal Surgery at the Hospital Emergencia Ate Vitarte and with approval from the Human Subjects Ethical Committee of the Administration of the Lima East Health System (DIRIS IV) – Ministry of Health (MINSA). This research has been reported in line with the STROCSS criteria [29] and has been registered through researchregistry.com with the unique identifying number (UIN), researchregistry6805 [30].

## 3 Results

58 patients, 35 male and 23 female, between ages 15 and 73, had principal symptoms: right iliac fossa abdominal pain (100%), focal rebound (98%), migration of pain (93%), nausea/vomiting (88%), leukocytosis (86%) and decreased appetite (84%). The average Alvarado Score was 8.1±1.1, the frequency of complicated appendicitis was 67% with the remainder uncomplicated. The appendicitis grade, according to the Gomes classification [14] was 19 (33%) grade I, 16 (28%) grade 2A, five (9%) grade 2B, two (3%) grade 3A, one (2%) grade 3B, three (5%) grade 3C and 12 (21%) grade 4.

Amidst the health crisis created by the pandemic, pre-existing diagnosis of COVID is mandatory for admission for our hospital. All included patients were sero-positive: seven (12%) via IgM only, 40 (69%) via IgM and IgG, and 11 (19%) IgG only (Table 1). Two patients were admitted to the hospital and underwent an operation, however one had indeterminate serologic status, and the other diagnosed via chest tomography alone, they were excluded from this study.

**Table 1:** Baseline characteristics of adults with SARS-CoV-2^a^ and acute appendicitis.

Sex [n^b^ (%)]		
Male	35	(60%)
Female	23	(40%)
**Age in years [Avg**^c^**(SD**^d^**)]**	34	(15.4)
**Nutritional State [n (%)]**		
Underweight	2	(3%)
Normal	16	(28%)
Overweight	32	(55%)
Class I Obesity	7	(12%)
Class 2 Obesity	1	(2%)
Class 3 Obesity	0	(0%)
**SARS-CoV-2 Diagnostic Method [n (%)]**		
IgM^e^	7	(12%)
IgM-IgG^f^	40	(69%)
IgG^g^	11	(19%)
**Appendicitis Category [n (%)]**		
Uncomplicated	19	(33%)
Complicated	39	(67%)
**Alvarado Score**		
Frequency of each component [n (%)]		
Pain in right iliac fossa	58	(100%)
Migration of pain	54	(93%)
Rebound	57	(98%)
Fever	11	(19%)
Decreased appetite	49	(85%)
Nausea/vomiting	51	(88%)
Leukocytosis	50	(86%)
Increased mature neutrophils (left shift)	34	(59%)
Total score [Avg (SD)]	8.1	(1.1)

a Coronavirus Disease 2019.

b Number of respondents.

c Average.

d Standard deviation.

e Immunoglobulin M.

f Immunoglobulin M-Immunoglobulin G.

g Immunoglobulin G.

None of the studied patients presented with respiratory signs or symptoms. Two cases presented with abdominal pain and digestive symptoms, with intraoperative finding, according to the operating surgeon's assessment, of a normal appendix. These two patients were excluded from the study; pathology reports not available to our investigators due to hospital policy. Similarly, two patients were excluded after undergoing operation for suspected acute appendicitis, when they were both found to have a normal appendix, one with acute diverticulitis the other with a foreign body in the distal ileum.

All patients underwent an open appendectomy, the great majority (90%) under regional anesthesia alone. This was conducted in accordance with recommendations from the Spanish Society of Surgeons for surgical management of patients with COVID [4].

The average time spent between diagnosis at an outside health facility and admission to our facility was 24±17 h, time from admission to our facility to initiation of operative intervention was 3.2±2.6 h, operative time 54±25 min, duration of post-operative hospital stay was 2.5±1.5 days.

All patients were successfully reached for follow-up at the pre-determined timeline. Eight of 58 (14%) patients experienced some type of post-operative event or complication. Seven of these were superficial surgical site events (Dindo-Clavien Grade I), four of which were abscesses, three seromas, all opened at the bedside. One patient had an organ-space surgical site infection (Dindo-Clavien Grade II) and required re-admission and antibiotic therapy. All complications were diagnosed within 14 days post-operatively. Patients who sustained complications all had complicated appendicitis and were overweight or obese. No patients sustained severe complications or death (Table 2).

**Table 2:** Peri- and intra-operative characteristics of adults with SARS-Cov-2^a^ and acute appendicitis.

Gomes Classification [n^b^ (%)]		
I. Inflamed appendix	19	(33%)
II A. Segmental necrosis	16	(28%)
II B. Base necrosis	5	(8%)
III A. Flegmom	2	(3%)
III B. Abscess < 5 cm without free air	1	(2%)
III C. Abscess > 5 cm without free air	3	(5%)
IV. Perforated - diffuse peritonitis±free air	12	(21%)
**Operative approach [n (%)]**		
Appendectomy	50	(86%)
Appendectomy with exploratory laparotomy	8	(14%)
**Drain placement [n (%)]**	14	(24%)
**Anesthesia Type [n (%)]**		
Regional	52	(90%)
General	6	(10%)
**Pre-hospital/transfer time in hours [Avg**^c^**(SD**^d^**)]**	24.2	[17]
**Time to operating room after arrival in hours [Avg (SD)]**	3.2	(2.6)
**Operative time in minutes [Avg (SD)]**	53.9	(25.3)
**Duration of post-operative hospitalization in days [Avg (SD)]**	2.5	(1.5)
**Complications (Dindo-Clavien Classification) [n (%)]**		
I. Deviation from normal post-operative course without need for additional pharmacologic or surgical intervention	7	(12%)
II. Deviation requiring antibiotics, transfusions	1	(2%)
III–V. Additional surgery, advanced therapy, death	0	(0%)
Total	8	(14%)
**Total subjects**	**58**	**100%**

a Coronavirus Disease 2019.

b Number of respondents.

c Average.

d Standard deviation.

## 4 Discussion

These data, in contrast to those presented in other reports [[5], [6], [7], [8]], reveal that with early open surgical intervention and regional anesthesia [12] patients with confirmed SARS-CoV-2 and appendicitis have a low rate of complications. All complications were minor (Dindo-Clavien I and II) with no associated mortality [20]. Eight patients (14%) had surgical site infections, seven superficial, one organ-space, all managed without re-operation and all in patients with complicated appendicitis and elevated body-mass index.

Acute appendicitis is the most common emergency general surgery pathology. Surgical management is ultimately recommended for complicated presentations [16], however, there is growing evidence of favorable outcomes with antibiotics alone for uncomplicated appendicitis [21]. Failure of non-operative management may present early, with 12% requiring operation within seven days, or late, with reports of 14–22% failure appreciated at one year [21,22]. A Cochrane systematic review from 2011 concluded that appendectomy continues to be the standard of care for patients with acute appendicitis, and that non-operative management may be an acceptable alternative in specific patients or in those with a contraindication to surgery [23].

At the onset of the SARS-CoV-2 pandemic the American College of Surgeons and the Association of Spanish Surgeons recommended non-operative management of patients with acute appendicitis and COVID-19, especially patients with uncomplicated appendicitis [4,10]. The non-operative approach was further delineated with a practical guide offered in a review by French surgeons published early in the health crisis [11]. However, in Latin American countries with low or middle-income classification like ours, non-operative management is difficult to institute safely, given the limited local experience, access to hospital beds, and anticipated difficulties with outpatient follow-up. A recently published multi-national study regarding laparoscopy for acute appendicitis included data from Peru and other low and middle-income countries. The authors cautioned: “when a healthcare system is struggling to deliver basic surgical procedures, the introduction of a more complex intervention must be considered carefully” [24]. We propose that amidst the COVID-19 pandemic and the associated health system collapse, non-operative management of acute appendicitis, which is not routine in Latin America, would require development of new processes, in particular with regard to patient follow-up, and would be associated with unacceptably high risk.

Recommendations for non-operative management of uncomplicated acute appendicitis in patients with COVID-19 have likely been encouraged by poor peri-operative outcomes in this population [11]. Exceptionally high morbidity and mortality associated with surgical management in patients with SARS-CoV-2 has been reported in countries such as Iran [5], China [6], Italy [7] and in a collaborative multi-national report [8]. In contrast, our report reveals a low rate of only minor post-operative complications (14%), and no deaths, in patients without respiratory symptoms with pre-operative diagnosis of COVID-19 and acute appendicitis undergoing open appendectomy. This indicates that open appendectomy is feasible in patients with SARS-CoV-2, but even more, that it may be a safe alternative as compared to laparoscopic appendectomy with regard to the frequency and grade of complications [25]. The high rates of respiratory complications and death which were reported in the largest multi-national study but not present in our experience, may be hypothesized to be due to our frequent (90%) use of regional anesthesia, however anesthetic approach was not associated with pulmonary or other complication in existing reports [8]. We recommend and are pursuing additional investigations to better understand what may account for the large variability in reported outcomes in SARS-CoV-2 patients, including operative and anesthetic approach, recognizing that the literature on the subject is immature and we currently can only conjecture.

The average pre-operative, operative, and admission duration were within the expected range as experienced by the authors at similar public hospitals within our system, and as compared to published reports [26]. Delay in operative intervention did occur due to the time spent after diagnosis at an outside hospital, prior to transfer to our institution. Dedication of our institution to the singular care of patients with SARS-CoV-2, and thus the presence of the necessary protocols and equipment, aided in us achieving early operative intervention [10], on average within 3.2 h in patients with acute appendicitis. It is our interpretation that the post-operative stay of the patient, on average 2.5 days, was not hindered or accelerated by SARS-CoV-2 diagnosis. Patients were neither discharged early to prevent exposure of other patients nor kept longer due to alteration in access to care or other resources.

Patients included in this study were seropositive for SARS-CoV-2 (IgG and/or IgM), which indicates contracting the virus approximately 10–14 days prior [27]. It does not indicate development of symptoms of COVID-19. All study patients were without respiratory symptoms, which may also be an explanation for the exceptionally low rate of morbidity and mortality in our cohort as compared to other published reports [[5], [6], [7], [8]].

This study is primarily limited by dependence on published reports for a comparison group, and lack of randomization of different therapeutic groups, in this case, primarily relevant to anesthetic approach. Efforts were made to collect data from a similar cohort without SARS-CoV-2 infection, however, given the current crisis of resources, and the fact that these patients would need to be from a separate institution, this did not prove feasible. We did not deem randomization to therapy acceptable given our concerns regarding the risk of introducing an untried approach, i.e. non-operative management, during a time of unparalleled health system strain. However, in this report the rate of surgical site infection, our only complication, was within the range reported in prior meta-analyses of open appendectomy [26]. As mentioned, the rate of any complication was significantly lower than that reported in multi-national reports of patients with SARS-CoV-2 [8]. A further limitation is the lack of access to SARS-CoV-2 PCR testing, which could have allowed for better delineation between active and previous SARS-CoV-2 infection. While this study was conducted early during the pandemic in Peru, it cannot be determined which serologically positive patients would also have been PCR positive. The unique strengths of this study include the exceptional 100% follow-up completion, which we attribute to increased patient responsiveness during a time when our population is gripped with fear regarding negative health outcomes. Furthermore, this report reflects a unique opportunity globally, as our hospital is one of few, and the only we're aware of within Latin America, to be opened exclusively for the treatment of patients who are SARS-CoV-2 positive [3].

In this study, two patients presented with gastrointestinal symptoms and abdominal pain and were intraoperatively found to have normal appendices without other surgical source of pain. We recommend the use of chest computed tomography (CT) [28] and abdominal CT with contrast in clinically indeterminate cases, those with a low Alvarado score [12] and in patients over age 60 according to the recommendation of the WSES Jerusalem Consensus [16].

## 5 Conclusion

These data demonstrate that early open surgical management of acute appendicitis in adults with pre-operative diagnosis of SARS-CoV-2 is feasible and is not associated with an increased frequency or severity of post-operative complications, longer operative time, or extended hospitalization as compared to reports in similar patients without SARS-CoV-2.

In low and middle-income countries, such as Peru, we recommend early open appendectomy under regional anesthesia as a safe and feasible option in adults with acute appendicitis and infection with SARS-Cov-2. Furthermore, we recommend cautious generalization of existing reports which suggest higher perioperative mortality in patients with SARS-CoV-2, as these data may not prove relevant to all or even most surgical patients, in particular as health systems mature in their ability to respond to, and cope with, the pandemic.

## Ethical approval

The study was authorized by the Hospital Administration within the project: Results of Emergency Abdominal Surgery at the Hospital Emergencia Ate Vitarte and with approval from the Human Subjects Ethical Committee of the Administration of the Lima East Health System (DIRIS IV) – Ministry of Health (MINSA).

## Funding

This research did not receive any specific grant from funding agencies in the public, commercial, or not-for-profit sectors.

## Author contribution

Eduardo Huamán Egoávil, MD MSc study design, data collection, data analysis, writing. Lacey LaGrone, MD MPH MA data analysis, writing. Regina Ugarte Oscco, MD (^1^) study design, data collection, data analysis, writing. Sergio Endo Ramos, MD (^1^) study design, data collection, data analysis, writing. Alex Diaz Baltazar, MD (^1^) study design, data collection, data analysis, writing. César Vergel Cabrera, MD (^4^) data analysis, writing.

## Conflict of interest

There were no conflicts of interest for this research study.

## Research registration unique identifying number (UIN)

Name of the registry: https://www.researchregistry.com/

Unique Identifying number or registration ID: researchregistry6805.

Hyperlink to your specific registration (must be publicly accessible and will be checked): https://www.researchregistry.com/browse-the-registry#home/

## Guarantor

Dr. Lacey LaGrone. 2500 Rocky Mountain Ave., Suite 2200 Loveland, CO 80538. Phone number: (206) 349–8249. Fax: 970.203.7526. lacey.lagrone@gmail.com.

## Provenance and peer review

Not commissioned, externally peer-reviewed.

## Declaration of competing interest

None.
